# Comparative Effectiveness of Bone Grafting Using Xenograft Freeze-Dried Cortical Bovine, Allograft Freeze-Dried Cortical New Zealand White Rabbit, Xenograft Hydroxyapatite Bovine, and Xenograft Demineralized Bone Matrix Bovine in Bone Defect of Femoral Diaphysis of White Rabbit: Experimental Study In Vivo

**DOI:** 10.1155/2017/7571523

**Published:** 2017-09-28

**Authors:** Ferdiansyah Mahyudin, Dwikora Novembri Utomo, Heri Suroto, Tri Wahyu Martanto, Mouli Edward, Imelda Lumban Gaol

**Affiliations:** Department of Orthopaedics and Traumatology, Airlangga University, Dr. Soetomo General Hospital, Surabaya, Indonesia

## Abstract

Autogenous bone graft is gold standard in treating bone defects, but it might have difficulty in corporation and rejection reaction. This study is to compare the effectiveness among freeze-dried xenograft, freeze-dried allograft, hydroxyapatite xenograft, and demineralized bone matrix xenograft as bone graft to fill bone defect in femoral diaphysis of white rabbit. Thirty male New Zealand white rabbits were distributed into five groups. Bone defect was filled correspondingly with xenograft freeze-dried cortical bovine, allograft freeze-dried cortical New Zealand white rabbit, xenograft hydroxyapatite bovine, and xenograft demineralized bone matrix bovine. No graft was used in control group. VEGF, osteoblast, and woven bone were higher in allograft freeze-dried cortical New Zealand white rabbit (mean 5.6625 (*p* < 0.05)) and xenograft demineralized bone matrix bovine (mean 5.2475 (*p* < 0.05)) with calcification of woven bone was already seen in week 2 in the latter group. There was a decrease of woven bone (mean 4.685 (*p* < 0.05)) fibrous tissue (mean 41.07 (*p* < 0.05)) in xenograft demineralized bone matrix bovine. The Immunoglobulin-G was elevated in control and all study groups but not significantly (*p* = 0.07855). Bone healing process in xenograft demineralized bone matrix bovine is more effective than in xenograft hydroxyapatite bovine, allograft freeze-dried New Zealand white rabbit, xenograft freeze-dried cortical bovine, and control.

## 1. Introduction

**Figure 1:**
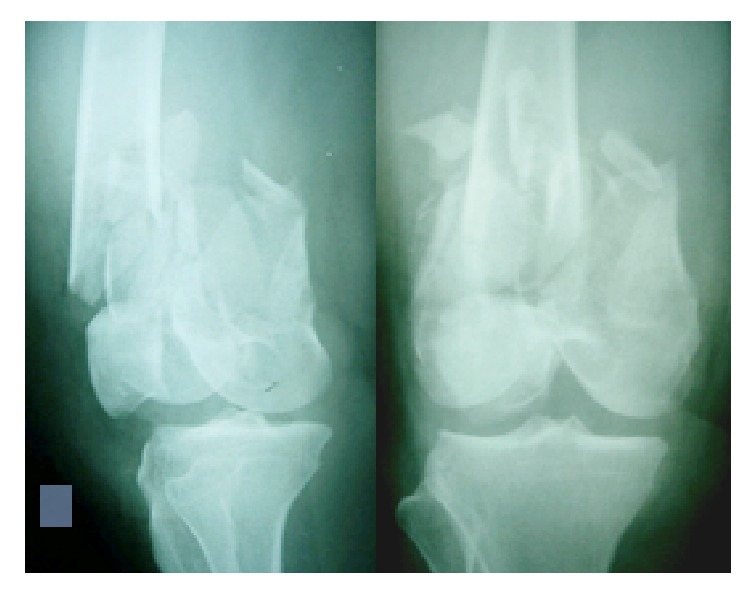
Bone defect after trauma.

Bone defects as seen in Figure 1 often occur due to trauma, tumor, osteitis, implant loosening, or corrective osteotomy. These bone defects should be filled with bone grafts to “bridge” the defects [1, 2]. Nowadays, bone graft transplantation is the second most frequently performed procedure after blood transfusion [3]. However, managing bone defects is a challenging problem as it requires high cost since there has been increase of demand due to the high number of operations worldwide (up to four millions operations a year) which requires the use of bone grafts [4]. In Indonesia, the need of bone grafts has begun to increase as there is an increase of trauma cases with bone defects and tumor resection cases that require bone graft to cover the defects. Based on the data from Tissue Bank of Dr. Soetomo General Hospital, the use of bone graft increased from 62 uses in 2010 to 75 and 178 uses in 2011 and 2012, respectively [5].

Autogenous bone graft becomes the gold standard because its ability of osteoinduction, osteoconduction, and osteogenesis and becomes the first choice of various experts and orthopaedic surgeons [6–8]. However, this autogenous bone graft has disadvantages of pain at the donor site and the potential occurrence of local complications such as hematoma, fractures, and the limited availability [7–9]. Due to the high morbidity and limited availability of autologous bone graft, it is considered to find a replacement with bone allograft [4].

Bone allografts have long been used as a natural substitute to cover the bone defect [4]. The advantage of the allograft is that the graft size can be adjusted. However it also has the disadvantages such as the transmission of diseases, rejection reactions, graft-host junction nonunion, graft resorption, fracture of allograft, and limitations of donor [6, 9, 10]. Later on, xenograft was found, in which functionality and complications are similar to allograft [9]. In the manufacturing process, freeze-drying causes the greatest reduction in bone strength; thus the tested method from Professor Frank Dexter of Tissue Bank Yorkshire has been chosen, which has been adapted by various tissue banks in Asia Pacific. However, the bone strength will still be reduced [11].

Shortage of freeze-dried allograft and freeze-dried xenograft encouraged the creation of hydroxyapatite (HA). The most unique nature of the HA material is its similarity to the mineral phase of bone. This similarity causes excellent osteoconductive capabilities and biocompatibility. HA is also regarded as the carrier of osteoinductive growth factors and good osteogenic cell population which is added to the HA since HA also functions as an introduction to transport bioactive [8]. HA has a high mechanical strength and biodegradability but has the disadvantages such as little axis and the toxic degradation product [3]. All these limitations of bone grafts resulted in the invention of demineralized bone matrix that also has osteoconductive and osteoinductive properties [12].

Demineralized bone matrix (DBM) is an allogenic bone that has been processed with pulverizing and decalcification using hydrochloric acid. The results are the loss of mineral components with retention of type I collagen, a noncollagenous protein, and osteoinductive growth factors, including varying concentrations of bone morphogenetic proteins (BMPs), growth differentiation factors, and possibility of transforming growth factors (TGF-b1, TGF-b2, and TGF-b3). With this content, DBM has osteoconductive and osteoinductive properties. DBM is touted as an excellent graft material for filling bone defects, nonunion of long bone, acute bone defect due to fractures, and spinal surgery procedures. DBM osteoconductive ability has been regarded as a result of mobilization from variety of growth factors contained within the extracellular matrix during demineralization process, thus making the potent bioactive molecules available in host environment. Induction often occurs due to interaction between osteoconductive factors and stem cells. The osteoconductive factors released by the donor bone that has been demineralized are considered to stimulate the existing stem cells to undergo osteogenic differentiation, resulting in tissue transformation and thus helping bone healing [12].

In bone healing, Vascular Endothelial Growth Factor (VEGF) is required to form the microvascular. With the presence of microvascular, phagocytosis by osteoclast is expected to occur more quickly followed by an increase in osteoblast which will accelerate the formation of woven bone and fibrous tissue degradation [12]. However, the provision of allograft or xenograft was feared to produce a rejection reaction as the immunological reactions [13]. It became a consideration by the expert in selecting the use of orthopaedic graft. Based on these circumstances, authors conducted a study on the effectiveness of bone healing on freeze-dried bovine, freeze-dried allograft, hydroxyapatite bovine, and demineralized bone matrix bovine to fill small bone defects in diaphysis.

## 2. Materials and Methods

Randomized posttest only control group design was performed on 30 male New Zealand white rabbits, weighing around 2.5–3.0 kg. All rabbits are anesthetized with intramuscular ketamine (40 mg/kg) and xylazine (13 mg/kg). Skin incision was done in the anterior side, 1 cm from the lateral epicondyle of the femur (diaphysis). The periosteum was also opened. Bone defects were made with diameter of 2.5 mm and depth of 2 mm (to penetrate the medulla). All rabbits were randomly and evenly divided into fifteen groups. The defects were filled with freeze-dried cortical bovine, freeze-dried cortical New Zealand white rabbit, bovine hydroxyapatite, and demineralized bone matrix bovine in which three groups were assigned for each material. The remaining three groups were the control group. After implantation, the periosteum and subcutaneous tissue were sutured with 2-0 absorbable thread. The skin was closed with 3-0 absorbable thread and covered with waterproof plaster. Cephazolin was given through intramuscular injections during surgery until 3 days after surgery.

Immunohistochemistry evaluation was conducted at weeks 1, 2, and 4 in control and study groups. Hematoxylin and eosin staining was performed for histological evaluation and examined by light microscope using two independent observers. Rejection reactions were checked by counting the amount of Immunoglobulin-G in serological reactions and then the amount of microvascular, osteoclasts, osteoblasts, bone, and broad woven fibrous tissue surrounding the graft at 100x and 400x magnification for histopathological examination and also VEGF and Immunoglobulin-G for immunohistochemistry examination was calculated.

## 3. Results

**Figure 2:**
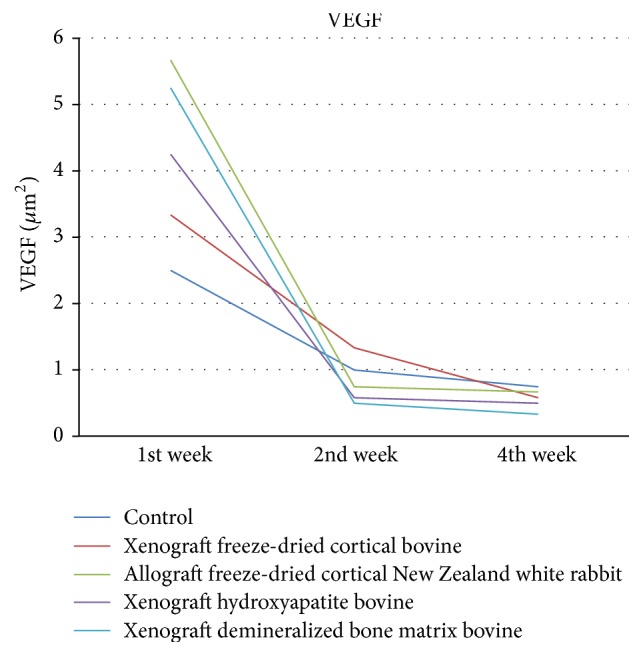
Evaluation of VEGF/*μ*m^2^.

Vascular Endothelial Growth Factor was high in all groups and decreased in weeks 2 and 4 with the highest number found in allograft freeze-dried cortical New Zealand white rabbit group, with no significant differences between allograft freeze-dried cortical New Zealand white rabbit and demineralized bone matrix bovine groups (*p* = 0.1) as shown in Figure 2.

**Figure 3:**
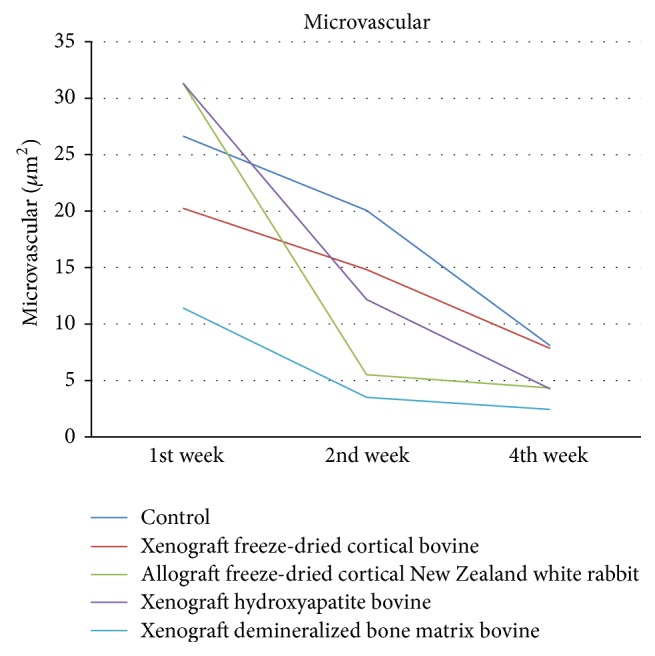
Evaluation of microvascular/*μ*m^2^.

Microvascular shown in Figure 3 was high in week 1 and decreased in weeks 2 and 4 in all groups, with the highest in allograft freeze-dried cortical New Zealand white rabbit group. There was a significant difference in demineralized bone matrix bovine group in week 1 (*p* = 0.02) and week 4 (*p* = 0.01), but not significant in week 2 (*p* = 1).

**Figure 4:**
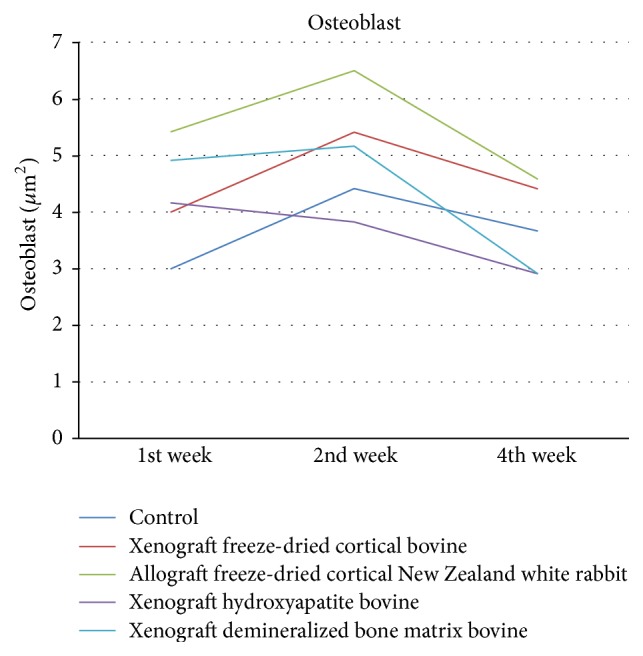
Evaluation of osteoblast/*μ*m^2^.

Osteoblast shown in Figure 4 showed significant difference in all groups in weeks 1, 2, and 4. It also obtained the highest increase in osteoblast in week 2 in all groups except control group. Osteoblast in allograft freeze-dried cortical New Zealand white rabbit group is higher than demineralized bone matrix bovine group. It was not significant in week 1 (*p* = 1), but it was significant in week 2 (*p* < 0.05) and week 4 (*p* = 0.001).

**Figure 5:**
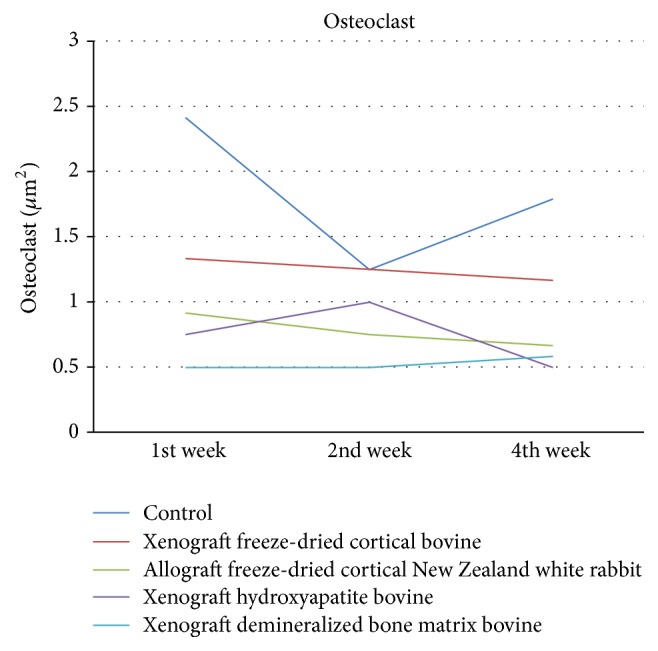
Evaluation of osteoclast/*μ*m^2^.

Osteoclast shown in Figure 5 showed significant differences in week 1 in all groups (*p* < 0.05) and also in week 4 in control, bovine hydroxyapatite, and freeze-dried cortical bovine groups (*p* < 0.05). The highest number of osteoclasts obtained was in control group in week 1. There was no significant differences between demineralized bone matrix bovine and freeze-dried cortical bovine group in week 1 (*p* = 0.018).

**Figure 6:**
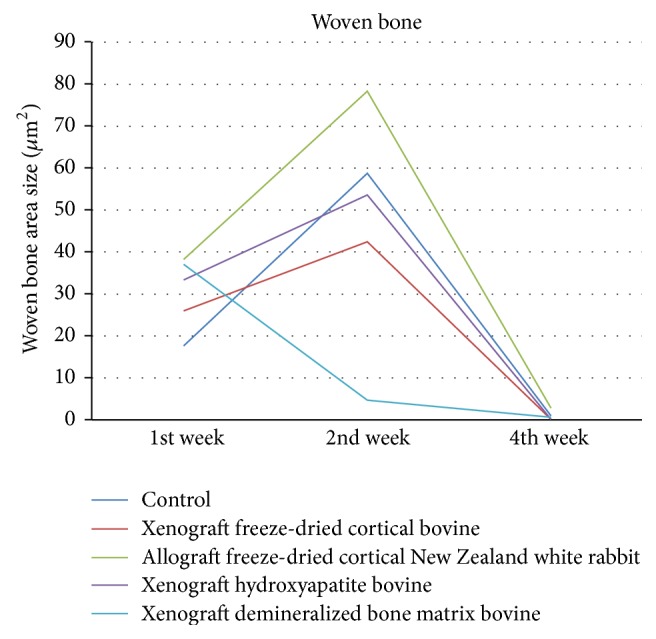
Evaluation of woven bone area size.

Woven bone shown in Figure 6 showed significant differences in week 2 between demineralized bone matrix bovine and all groups (*p* < 0.05). There were increases in all groups except for demineralized bone matrix bovine group in week 2. Woven bone in demineralized bone matrix bovine decreased in weeks 2 and 4.

**Figure 7:**
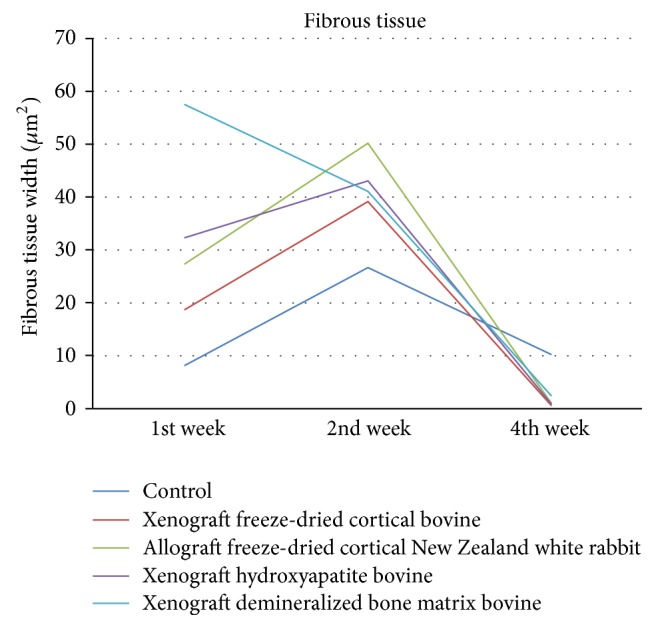
Evaluation of fibrous tissue width (*μ*).

Figure 7 showed significant differences in the mean width of fibrous tissue of all groups at weeks 1, 2, and 4. The width increase in fibrous tissue was shown in week 2 and decreased in week 4 in all groups except for demineralized bone matrix bovine group. Demineralized bone matrix bovine decreased in weeks 2 and 4. There was a significant difference between all groups in week 1 (*p* < 0.05), but no significant difference in weeks 2 and 4 (*p* > 0.05).

**Figure 8:**
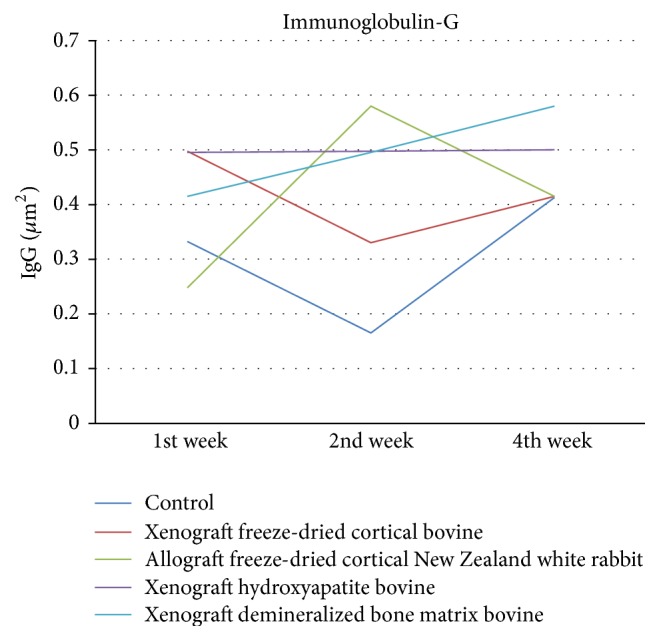
Evaluation of IgG/*μ*m^2^.

There is no significant differences for Immunoglobulin-G in all groups in week 1 (*p* > 0.05), week 2 (*p* > 0.05), and week 4 (*p* > 0.05) as shown in Figure 8.

**Figure 9:**
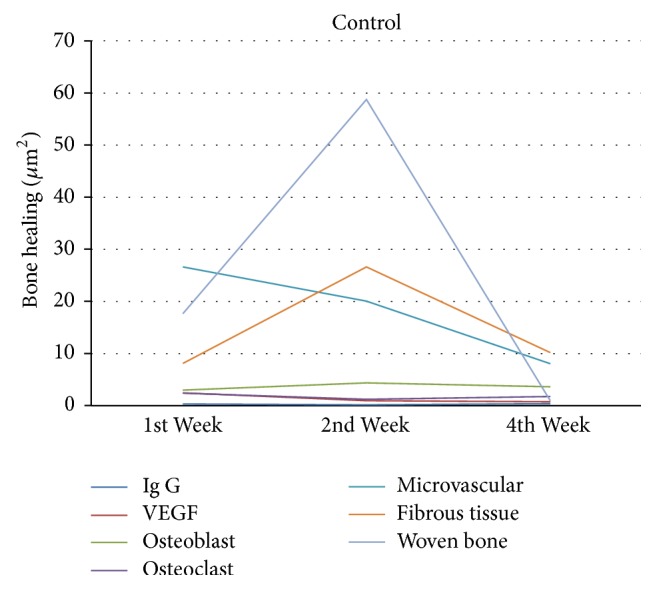
Bone healing in control group.

In control group as shown in Figure 9, there was an increase of VEGF in week 1 followed by an increase of microvascular and osteoclast formation. The width of fibrous tissue was high, while that of woven bone was less. VEGF formation decreased in week 2, accompanied by decrease of microvascular and osteoclast formation. Osteoblast increased along with woven bone and fibrous tissue formation. The number of VEGF was less in week 4 accompanied by decreasing microvascular, osteoblast, and woven bone, while osteoclast increased along with fibrous tissue. Immunoglobulin-G decreased in week 2 and increased in week 4.

**Figure 10:**
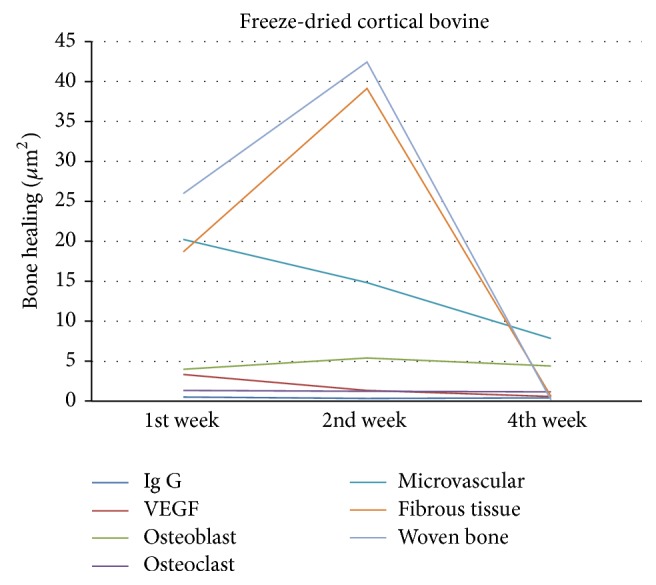
Bone healing in freeze-dried cortical bovine group.

In freeze-dried cortical bovine group as shown in Figure 10, there was an increase in VEGF in week 1 accompanied by increasing microvascular and osteoclast formation and caused phagocytosis by osteoclasts. The width of fibrous tissue was high, while that of woven bone was less. VEGF formation decreased in week 2, accompanied by decreasing microvascular and osteoclast formation. Osteoblast increased along with woven bone and fibrous tissue formation. VEGF in week 4 was very low with microvascular dwindling. Osteoclast and osteoblast decreased and woven bone formed very slightly and turned into lamellar bone with less number of fibrous tissues. Immunoglobulin-G decreased in weeks 2 and 4 but not significantly.

**Figure 11:**
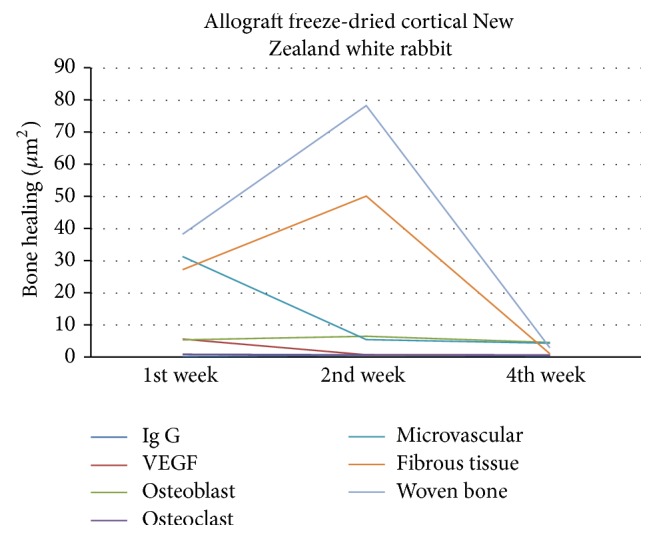
Bone healing in allograft freeze-dried cortical New Zealand white rabbit group.

In allograft freeze-dried cortical New Zealand white rabbit group as shown in Figure 11, VEGF increased in week 1 along with the high microvascular and osteoblasts and began to increase the formation of woven bone and fibrous tissue. VEGF decreased in week 2 along with the number of microvascular and osteoclast, but the osteoblast increased. This was followed by the formation of woven bone and fibrous tissue which were the highest in week 2. VEGF, microvascular, osteoblast, osteoclast, and fibrous tissue decreased in week 4. Woven bone was already transformed into lamellar bone. Immunoglobulin-G was formed in week 1 and increased in week 2 but not significantly and then decreased in week 4.

**Figure 12:**
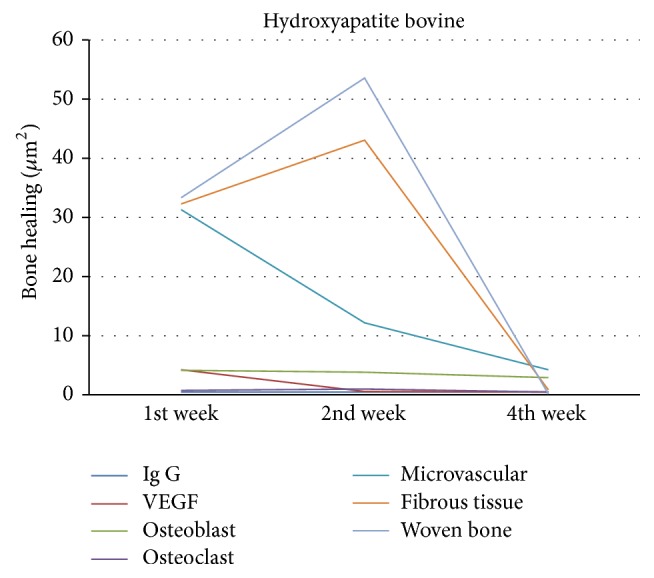
Bone healing in bovine hydroxyapatite group.

In bovine hydroxyapatite group as shown in Figure 12, VEGF increased in week 1 along with the high microvascular and osteoblasts and began to increase the formation of woven bone and fibrous tissue. VEGF decreased in week 2 along with the number of microvascular and osteoclasts, but osteoblast increased. This was followed by the formation of woven bone and fibrous tissue which were the highest in week 2. VEGF, microvascular, osteoblast, osteoclast, and fibrous tissue decreased in week 4. Immunoglobulin-G was formed in week 1 and increased in week 2, but not significantly, and then decreased in week 4. This is similar to allograft freeze-dried cortical New Zealand white rabbit group.

**Figure 13:**
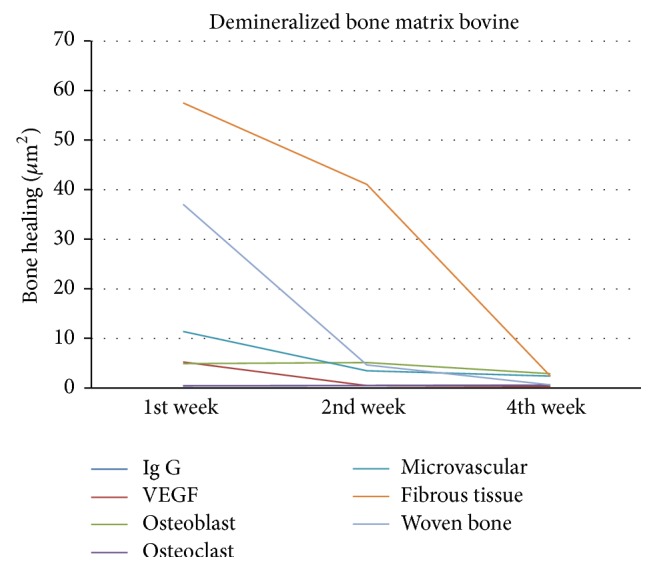
Bone healing in demineralized bone matrix bovine group.

In demineralized bone matrix bovine group as shown in Figure 13, VEGF obtained was the highest in week 1 followed by the number of microvascular, osteoclast, fibrous tissue, and woven bone. Osteoblast obtained was high in week 2. VEGF, microvascular, osteoclast, and fibrous tissue then decreased in week 4. Woven bone calcification decreased in week 2. All components significantly decreased in week 4. Immunoglobulin-G is high in week 1 and decreased in weeks 2 and 4.

**Figure 14:**
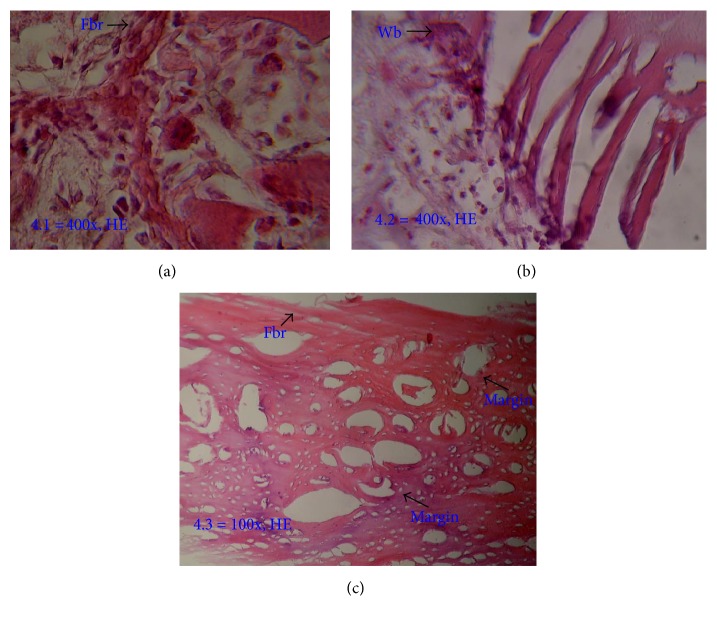
Histology of white rabbit's femur (diaphysis) in demineralized bone matrix bovine group. (a) In week 1, high amount of woven bone and fibrous tissue; (b) in week 2, decrease of fibrous tissue and woven bone; (c) in week 4, unclear margin between woven bone and native bone, representing calcified woven bone. Fbr: fibrous tissue; Wb: woven bone.

In demineralized bone matrix bovine group, osteoclast and osteoblast were present with high amount of woven bone and fibrous tissue in week 1, as histologically shown in Figure 14(a). In the second week, there was decrease of fibrous tissue and woven bone, as well as osteoblast and osteoclast, due to process of calcification (as shown in Figure 14(b)). In week 4, the fibrous tissue and woven bone decreased with thin woven bone already calcified, as proven by unclear margin between woven bone and native bone, as shown in Figure 14(c).

**Figure 15:**
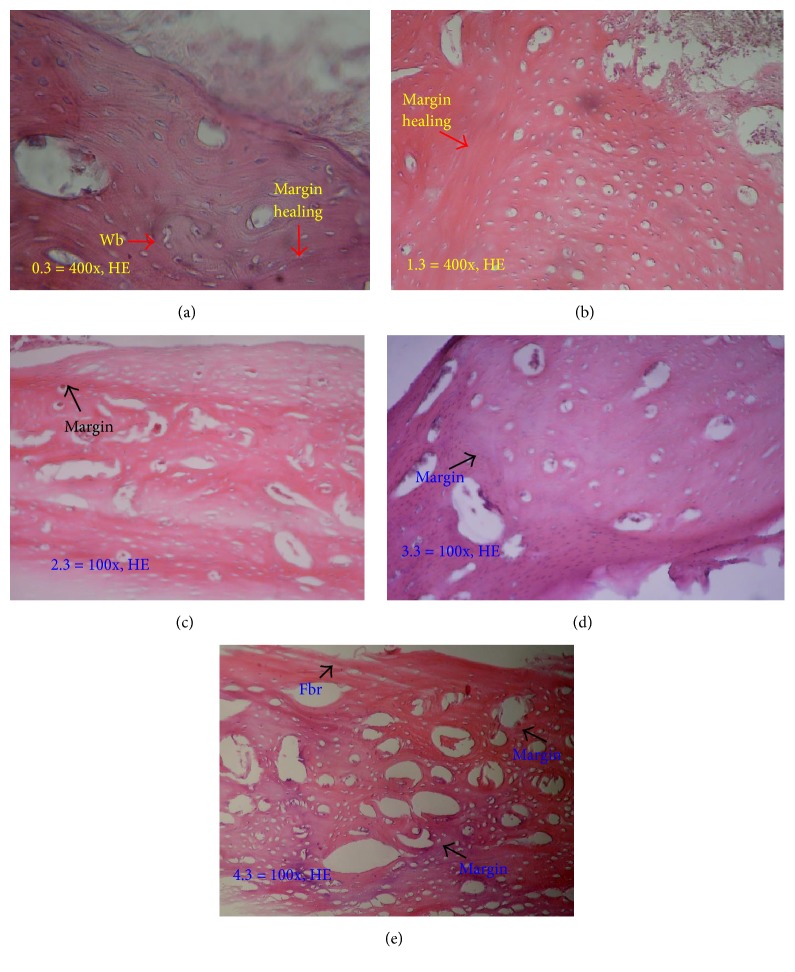
Histology in week 4. Clear margin between healing process and native bone shown in control group (a); freeze-dried bovine cortical group (b); allograft freeze-dried cortical New Zealand white rabbit group (c); and hydroxyapatite bovine group (d). In other hand, the healing in demineralized bone matrix bovine group (e) already resembles the native bone. Fbr: fibrous tissue.

Figure 15 shows difference in bone healing between demineralized bone matrix bovine group with other groups in week 4. The healing (arrow) in demineralized bone matrix bovine already resembles the original bone, such as bone in the margins, that is smaller and less porous, and fibrous tissue thin is not as thick as the other groups (Figure 15(e)), while, in other groups, we can clearly see the margin in the healing process with the native bone (Figures 15(a)–15(d)).

**Figure 16:**
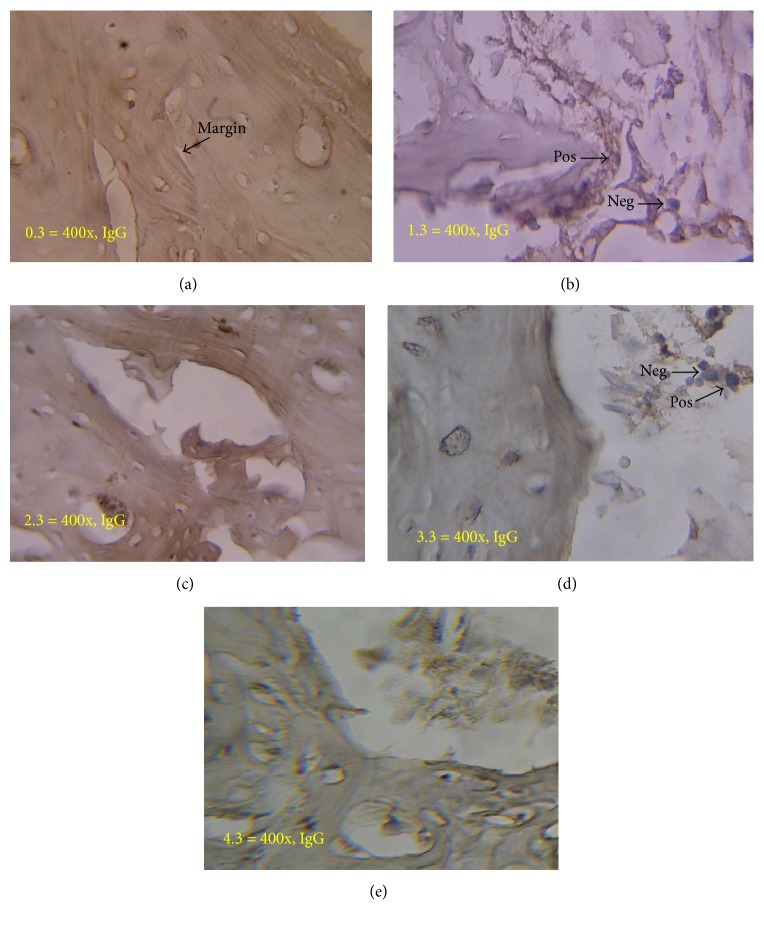
Immunology reaction in week 4. There is no difference in Immunoglobulin-G reaction in control group (a); freeze-dried bovine cortical group (b); allograft freeze-dried cortical New Zealand white rabbit group (c); hydroxyapatite bovine group (d); and demineralized bone matrix bovine group (e).

Figure 16 shows the reaction of Immunoglobulin-G in each group. There is no difference in Immunoglobulin-G reaction result of all groups in weeks 1, 2, and 4.

## 4. Discussion

In the evaluation of VEGF, VEGF formation was high on allograft demineralized bone matrix bovine and freeze-dried New Zealand white rabbit, but there was no difference between the groups in week 4. The highest microvascular increase was seen in week 1 in all groups. VEGF formation rise may be influenced by osteoinduction of demineralized bone matrix bovine. VEGF formation rise induces acceleration of angiogenesis. This is consistent with previous studies that VEGF stimulates bone repair in angiogenesis formation and bone turnover [14–16]. Osteoblast increased in week 2 in all groups except in hydroxyapatite bovine, in which the number of osteoclasts increased in week 2 and decreased in week 4. Woven bone was formed in week 1 and increased up to week 2 in all groups except in demineralized bone matrix bovine, whereby in this group the highest woven bone was found in week 1 and decreased in week 2 due to calcification. The process of callus formation will occur from the first week and will be maximum in second week along with calcification until healing occurs.

This study found no significant differences in bone healing process in week 4 in freeze-dried New Zealand white rabbit, freeze-dried cortical bovine, and hydroxyapatite bovine groups. This is in contrary with previous literature which stated that the allograft bone healing process was better than that of xenograft [17]. This can happen because of the process of making a good xenograft that allows the healing process of bone to resemble bone allograft. This study found that there was no difference in the healing process of bone hydroxyapatite compared with allograft. This is consistent with the previous studies [18].

Demineralized bone matrix bovine obtained the highest VEGF formation in week 1 in conjunction with the high amount of microvascular, osteoblast, osteoclast, and woven bone with fibrous tissue which has been narrowed compared to week 1. In the second week, the calcification of woven bone caused decrease in the amount of woven bone along with fibrous tissue.

From this study, the bone healing process in demineralized bone matrix bovine group occurred faster compared to other groups. This study agreed with previous studies which concluded that bone healing in the DBM is better than in HA [19].

Evaluation of Immunoglobulin-G found no significant differences in all groups. In control group, an increase in IgG showed that immunological reactions will still exist with no significant differences. This is different from previous studies which stated that immunological response occurs higher in the xenograft compared to the allograft [17]. Immunological reactions can be minimized with good processing techniques. In addition, immunological reactions on hydroxyapatite can be ignored [20], according to the results of this study.

## 5. Conclusion

Bone healing in demineralized bone matrix bovine group was more effective than in hydroxyapatite bovine, freeze-dried New Zealand white rabbit, freeze-dried cortical bovine, and control groups.

## Additional Points

*Future Work*. This research is aimed at searching for autologous bone graft's alternatives in treating certain orthopaedic cases. Autologous bone graft is still the gold standard with several limitations such as small amount of available source, difficulty in obtaining the desirable shape, and ultimately the potential unwanted donor site morbidity. These products, especially demineralized bone matrix from bovine bone, were researched in order to determine the best alternative for autologous bone graft, enabling mass production in the future. Future research for this material is to test the structural strength of the graft against compression and bending force, determining its efficacy in solving the bone defect problems. The treatment of nonunion fractures or large bone defects cases using xenografts has not yet produced satisfactory results. An ideal bone graft should innately possess osteogenesis, osteoinductive, and osteoconductive characteristics. To obtain all the characteristics of ideal bone graft, the current on-going and future researches are aimed at upgrading the efficacy of aforementioned products. The proposed method in those researches is the application of tissue engineering principle by seeding stem cells on the bone graft.
